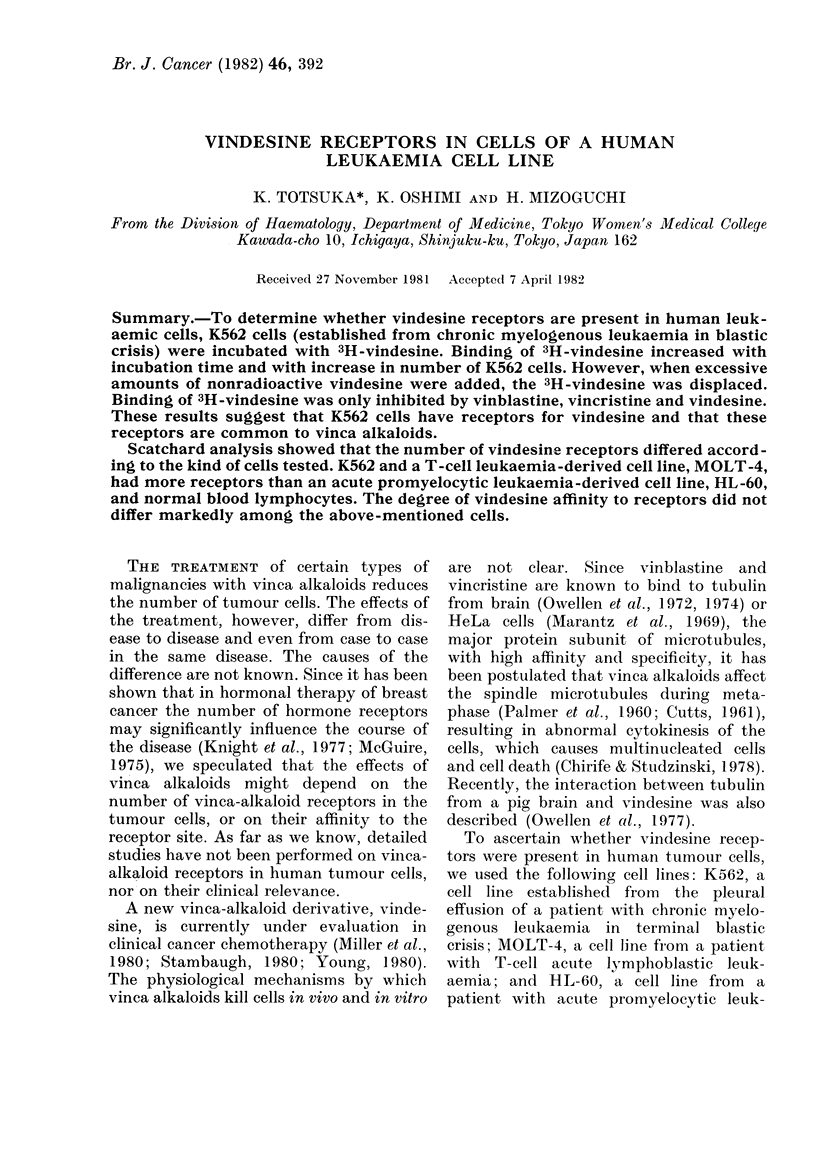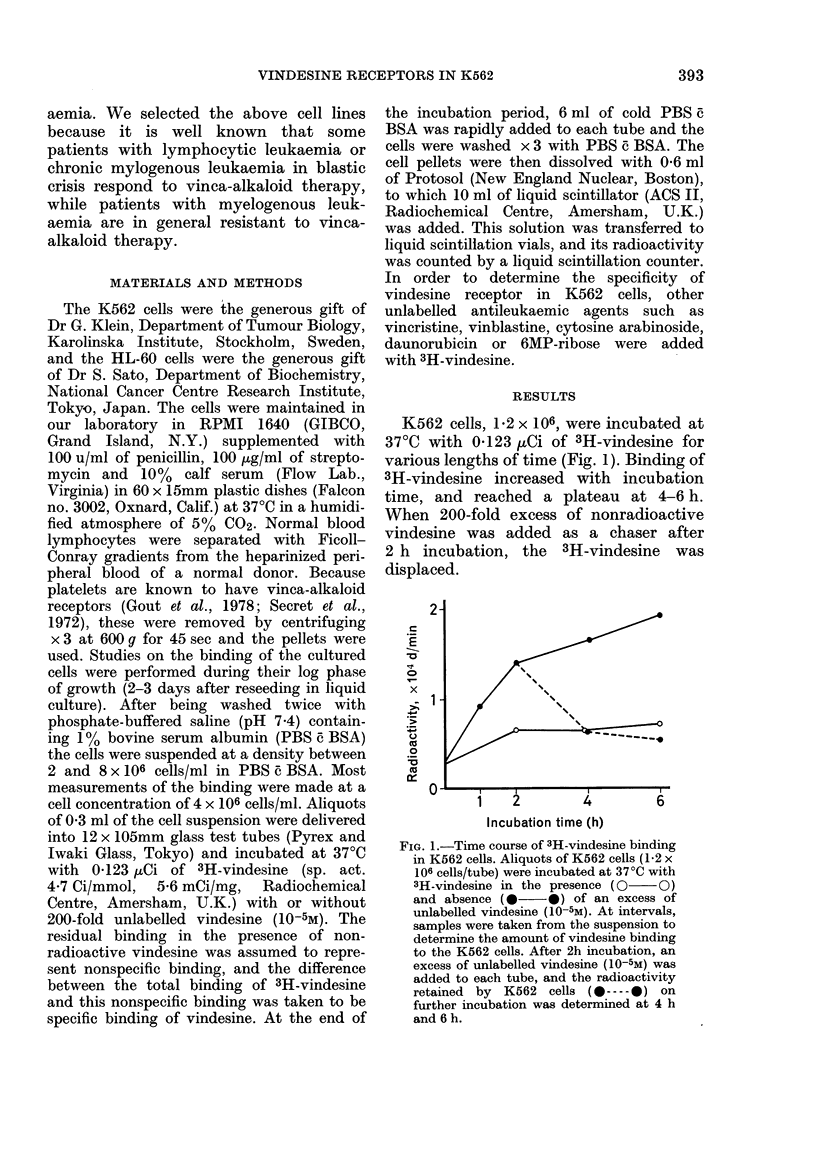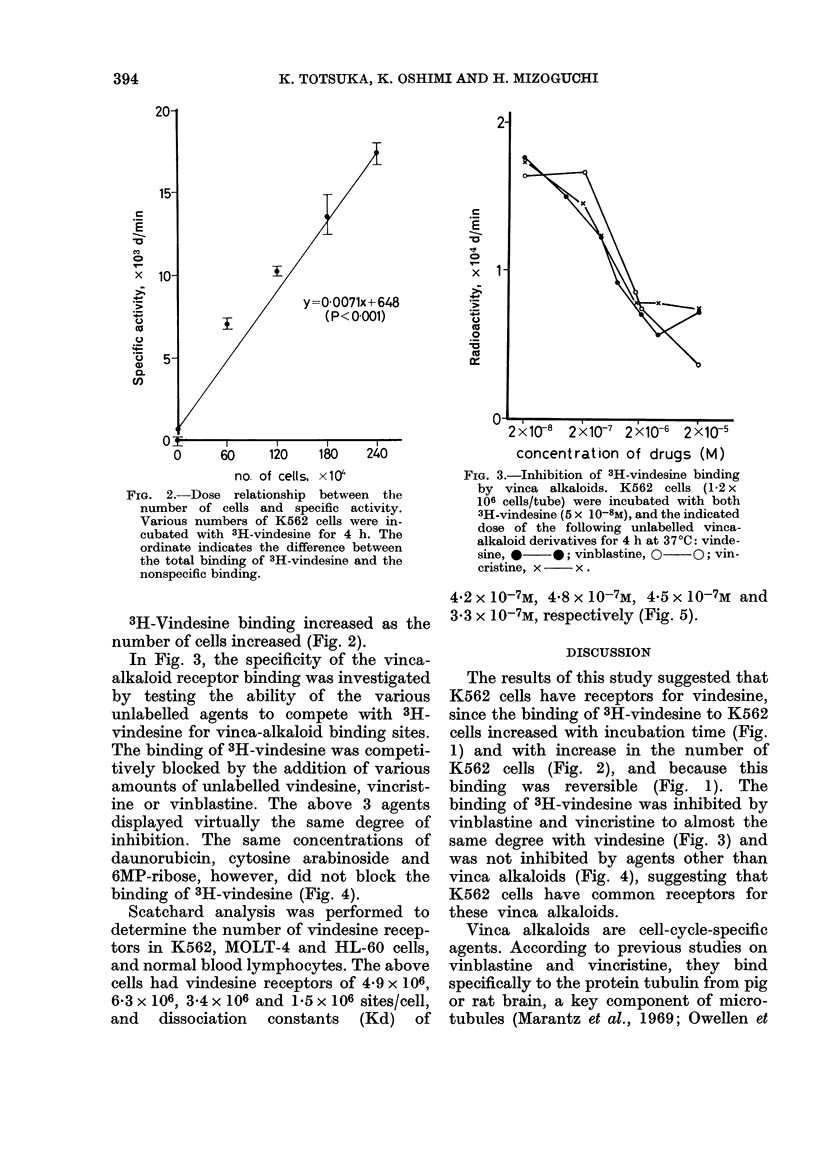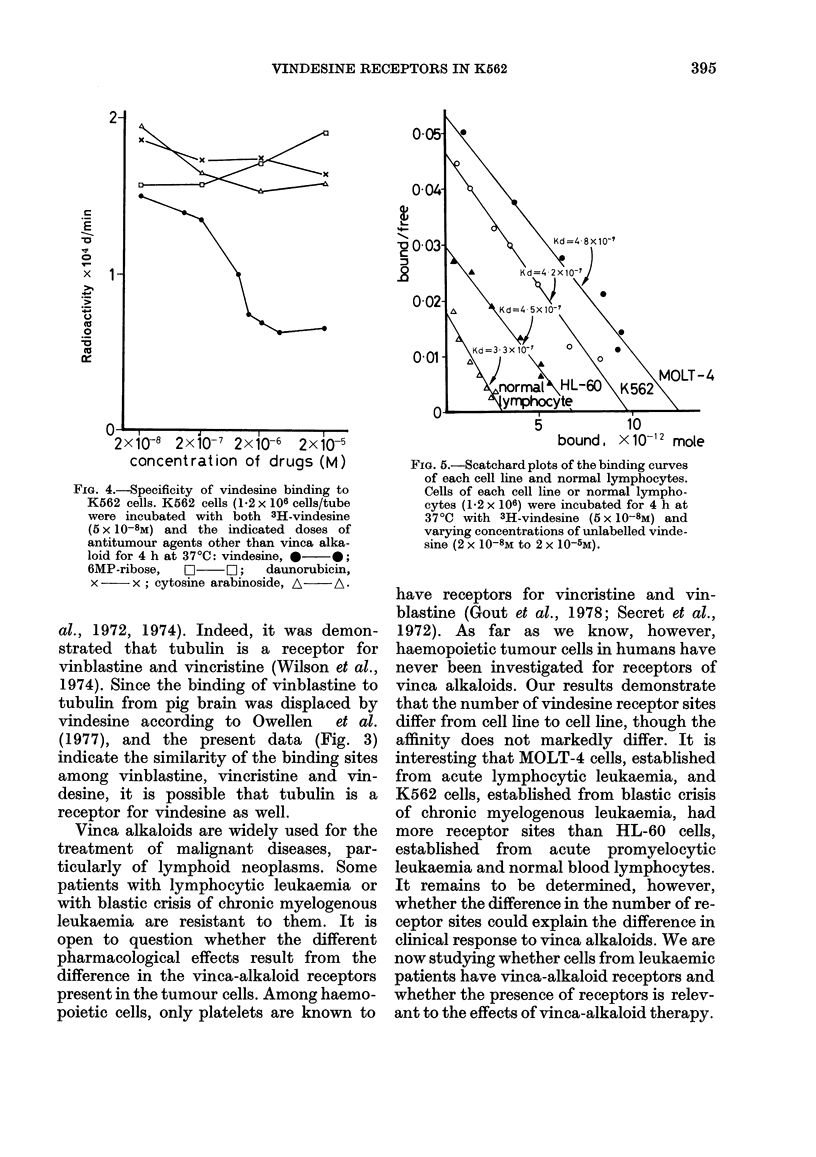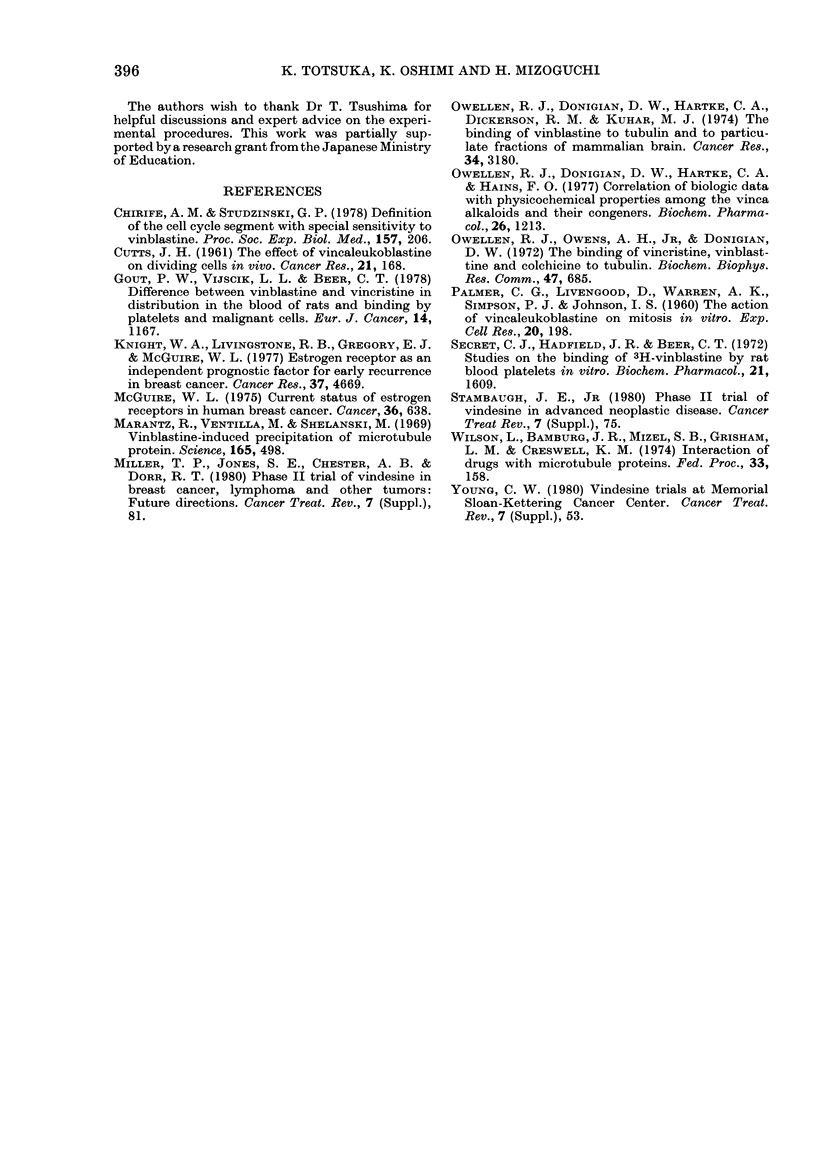# Vindesine receptors in cells of a human leukaemia cell line.

**DOI:** 10.1038/bjc.1982.215

**Published:** 1982-09

**Authors:** K. Totsuka, K. Oshimi, H. Mizoguchi

## Abstract

To determine whether vindesine receptors are present in human leukaemic cells, K562 cells (established from chronic myelogenous leukaemia in blastic crisis) were incubated with 3H-vindesine. Binding of 3H-vindesine increased with incubation time and with increase in number of K562 cells. However, when excessive amounts of nonradioactive vindesine were added, the 3H-vindesine was displaced. Binding of 3H-vindesine was only inhibited by vinblastine, vincristine and vindesine. These results suggest that K562 cells have receptors for vindesine and that these receptors are common to vinca alkaloids. Scatchard analysis showed that the number of vindesine receptors differed according to the kind of cells tested. K562 and a T-cell leukaemia-derived cell line, MOLT-4, had more receptors than an acute promyelocytic leukaemia-derived cell line, HL-60, and normal blood lymphocytes. The degree of vindesine affinity to receptors did not differ markedly among the above-mentioned cells.


*Br. J. Cancer* (1982) **46,** 392

### VINDESINE RECEPTORS IN CELLS OF A HUMAN LEUKAEMIA CELL LINE

K. TOTSUKA*, K. OSHIMI AND H. MIZOGUCHI

*From the Division of Haematology, Department of Medicine, Tokyo Women's Medical College Kawada-cho* 10, *Ichigaya, Shinjuku-ku, Tokyo, Japan* 162

Received 27 November 1981 Accepted 7 April 1982

**Summary.—To determine whether vindesine receptors are present in human leukaemic cells, K562 cells (established from chronic myelogenous leukaemia in blastic crisis) were incubated with $^{3}$H-vindesine. Binding of $^{3}$H-vindesine increased with incubation time and with increase in number of K562 cells. However, when excessive amounts of nonradioactive vindesine were added, the $^{3}$H-vindesine was displaced. Binding of $^{3}$H-vindesine was only inhibited by vinblastine, vincristine and vindesine. These results suggest that K562 cells have receptors for vindesine and that these receptors are common to vinca alkaloids.**

**Scatchard analysis showed that the number of vindesine receptors differed according to the kind of cells tested. K562 and a T-cell leukaemia-derived cell line, MOLT-4, had more receptors than an acute promyelocytic leukaemia-derived cell line, HL-60, and normal blood lymphocytes. The degree of vindesine affinity to receptors did not differ markedly among the above-mentioned cells.**

THE TREATMENT of certain types of malignancies with vinca alkaloids reduces the number of tumour cells. The effects of the treatment, however, differ from disease to disease and even from case to case in the same disease. The causes of the difference are not known. Since it has been shown that in hormonal therapy of breast cancer the number of hormone receptors may significantly influence the course of the disease (Knight *et al.*, 1977; McGuire, 1975), we speculated that the effects of vinca alkaloids might depend on the number of vinca-alkaloid receptors in the tumour cells, or on their affinity to the receptor site. As far as we know, detailed studies have not been performed on vinca-alkaloid receptors in human tumour cells, nor on their clinical relevance.

A new vinca-alkaloid derivative, vindesine, is currently under evaluation in clinical cancer chemotherapy (Miller *et al.*, 1980; Stambaugh, 1980; Young, 1980). The physiological mechanisms by which vinca alkaloids kill cells *in vivo* and *in vitro* are not clear. Since vinblastine and vincristine are known to bind to tubulin from brain (Owellen *et al.*, 1972, 1974) or HeLa cells (Marantz *et al.*, 1969), the major protein subunit of microtubules, with high affinity and specificity, it has been postulated that vinca alkaloids affect the spindle microtubules during metaphase (Palmer *et al.*, 1960; Cutts, 1961), resulting in abnormal cytokinesis of the cells, which causes multinucleated cells and cell death (Chirife & Studzinski, 1978). Recently, the interaction between tubulin from a pig brain and vindesine was also described (Owellen *et al.*, 1977).

To ascertain whether vindesine receptors were present in human tumour cells, we used the following cell lines: K562, a cell line established from the pleural effusion of a patient with chronic myelogenous leukaemia in terminal blastic crisis; MOLT-4, a cell line from a patient with T-cell acute lymphoblastic leukaemia; and HL-60, a cell line from a patient with acute promyelocytic leukaemia. We selected the above cell lines because it is well known that some patients with lymphocytic leukaemia or chronic mylogenous leukaemia in blastic crisis respond to vinca-alkaloid therapy, while patients with myelogenous leukaemia are in general resistant to vinca-alkaloid therapy.

### MATERIALS AND METHODS

The K562 cells were the generous gift of Dr G. Klein, Department of Tumour Biology, Karolinska Institute, Stockholm, Sweden, and the HL-60 cells were the generous gift of Dr S. Sato, Department of Biochemistry, National Cancer Centre Research Institute, Tokyo, Japan. The cells were maintained in our laboratory in RPMI 1640 (GIBCO, Grand Island, N.Y.) supplemented with 100 u/ml of penicillin, 100 $\mu$g/ml of streptomycin and 10% calf serum (Flow Lab., Virginia) in 60 x 15mm plastic dishes (Falcon no. 3002, Oxnard, Calif.) at 37°C in a humidified atmosphere of 5% $CO_2$. Normal blood lymphocytes were separated with Ficoll–Conray gradients from the heparinized peripheral blood of a normal donor. Because platelets are known to have vinca-alkaloid receptors (Gout *et al.*, 1978; Secret *et al.*, 1972), these were removed by centrifuging x 3 at 600 *g* for 45 sec and the pellets were used. Studies on the binding of the cultured cells were performed during their log phase of growth (2–3 days after reseeding in liquid culture). After being washed twice with phosphate-buffered saline (pH 7·4) containing 1% bovine serum albumin (PBS c̄ BSA) the cells were suspended at a density between 2 and $8 \times 10^6$ cells/ml in PBS c̄ BSA. Most measurements of the binding were made at a cell concentration of $4 \times 10^6$ cells/ml. Aliquots of 0·3 ml of the cell suspension were delivered into 12 x 105mm glass test tubes (Pyrex and Iwaki Glass, Tokyo) and incubated at 37°C with 0·123 $\mu$Ci of $^3$H-vindesine (sp. act. 4·7 Ci/mmol, 5·6 mCi/mg, Radiochemical Centre, Amersham, U.K.) with or without 200-fold unlabelled vindesine ($10^{-5}$M). The residual binding in the presence of nonradioactive vindesine was assumed to represent nonspecific binding, and the difference between the total binding of $^3$H-vindesine and this nonspecific binding was taken to be specific binding of vindesine. At the end of the incubation period, 6 ml of cold PBS c̄ BSA was rapidly added to each tube and the cells were washed x 3 with PBS c̄ BSA. The cell pellets were then dissolved with 0·6 ml of Protosol (New England Nuclear, Boston), to which 10 ml of liquid scintillator (ACS II, Radiochemical Centre, Amersham, U.K.) was added. This solution was transferred to liquid scintillation vials, and its radioactivity was counted by a liquid scintillation counter. In order to determine the specificity of vindesine receptor in K562 cells, other unlabelled antileukaemic agents such as vincristine, vinblastine, cytosine arabinoside, daunorubicin or 6MP-ribose were added with $^3$H-vindesine.

### RESULTS

K562 cells, $1{\cdot}2 \times 10^6$, were incubated at 37°C with 0·123 $\mu$Ci of $^3$H-vindesine for various lengths of time (Fig. 1). Binding of $^3$H-vindesine increased with incubation time, and reached a plateau at 4–6 h. When 200-fold excess of nonradioactive vindesine was added as a chaser after 2 h incubation, the $^3$H-vindesine was displaced.

FIG. 1.—Time course of $^3$H-vindesine binding in K562 cells. Aliquots of K562 cells ($1{\cdot}2 \times 10^6$ cells/tube) were incubated at 37°C with $^3$H-vindesine in the presence (○——○) and absence (●——●) of an excess of unlabelled vindesine ($10^{-5}$M). At intervals, samples were taken from the suspension to determine the amount of vindesine binding to the K562 cells. After 2h incubation, an excess of unlabelled vindesine ($10^{-5}$M) was added to each tube, and the radioactivity retained by K562 cells (●----●) on further incubation was determined at 4 h and 6 h.

FIG. 2.—Dose relationship between the number of cells and specific activity. Various numbers of K562 cells were incubated with $^{3}$H-vindesine for 4 h. The ordinate indicates the difference between the total binding of $^{3}$H-vindesine and the nonspecific binding.

FIG. 3.—Inhibition of $^{3}$H-vindesine binding by vinca alkaloids. K562 cells ($1{\cdot}2\times10^{6}$ cells/tube) were incubated with both $^{3}$H-vindesine ($5\times10^{-8}$M), and the indicated dose of the following unlabelled vinca-alkaloid derivatives for 4 h at 37°C: vindesine, ●——●; vinblastine, ○——○; vincristine, ×——×.

$^{3}$H-Vindesine binding increased as the number of cells increased (Fig. 2).

In Fig. 3, the specificity of the vinca-alkaloid receptor binding was investigated by testing the ability of the various unlabelled agents to compete with $^{3}$H-vindesine for vinca-alkaloid binding sites. The binding of $^{3}$H-vindesine was competitively blocked by the addition of various amounts of unlabelled vindesine, vincristine or vinblastine. The above 3 agents displayed virtually the same degree of inhibition. The same concentrations of daunorubicin, cytosine arabinoside and 6MP-ribose, however, did not block the binding of $^{3}$H-vindesine (Fig. 4).

Scatchard analysis was performed to determine the number of vindesine receptors in K562, MOLT-4 and HL-60 cells, and normal blood lymphocytes. The above cells had vindesine receptors of $4{\cdot}9\times10^{6}$, $6{\cdot}3\times10^{6}$, $3{\cdot}4\times10^{6}$ and $1{\cdot}5\times10^{6}$ sites/cell, and dissociation constants (Kd) of $4{\cdot}2\times10^{-7}$M, $4{\cdot}8\times10^{-7}$M, $4{\cdot}5\times10^{-7}$M and $3{\cdot}3\times10^{-7}$M, respectively (Fig. 5).

### DISCUSSION

The results of this study suggested that K562 cells have receptors for vindesine, since the binding of $^{3}$H-vindesine to K562 cells increased with incubation time (Fig. 1) and with increase in the number of K562 cells (Fig. 2), and because this binding was reversible (Fig. 1). The binding of $^{3}$H-vindesine was inhibited by vinblastine and vincristine to almost the same degree with vindesine (Fig. 3) and was not inhibited by agents other than vinca alkaloids (Fig. 4), suggesting that K562 cells have common receptors for these vinca alkaloids.

Vinca alkaloids are cell-cycle-specific agents. According to previous studies on vinblastine and vincristine, they bind specifically to the protein tubulin from pig or rat brain, a key component of microtubules (Marantz *et al.*, 1969; Owellen *et*

FIG. 4.—Specificity of vindesine binding to K562 cells. K562 cells ($1\cdot2 \times 10^6$ cells/tube were incubated with both $^3$H-vindesine ($5 \times 10^{-8}$M) and the indicated doses of antitumour agents other than vinca alkaloid for 4 h at 37°C: vindesine, ●——●; 6MP-ribose, □——□; daunorubicin, ×——× ; cytosine arabinoside, △——△.

FIG. 5.—Scatchard plots of the binding curves of each cell line and normal lymphocytes. Cells of each cell line or normal lymphocytes ($1\cdot2 \times 10^6$) were incubated for 4 h at 37°C with $^3$H-vindesine ($5 \times 10^{-8}$M) and varying concentrations of unlabelled vindesine ($2 \times 10^{-8}$M to $2 \times 10^{-5}$M).

*al.*, 1972, 1974). Indeed, it was demonstrated that tubulin is a receptor for vinblastine and vincristine (Wilson *et al.*, 1974). Since the binding of vinblastine to tubulin from pig brain was displaced by vindesine according to Owellen *et al.* (1977), and the present data (Fig. 3) indicate the similarity of the binding sites among vinblastine, vincristine and vindesine, it is possible that tubulin is a receptor for vindesine as well.

Vinca alkaloids are widely used for the treatment of malignant diseases, particularly of lymphoid neoplasms. Some patients with lymphocytic leukaemia or with blastic crisis of chronic myelogenous leukaemia are resistant to them. It is open to question whether the different pharmacological effects result from the difference in the vinca-alkaloid receptors present in the tumour cells. Among haemopoietic cells, only platelets are known to have receptors for vincristine and vinblastine (Gout *et al.*, 1978; Secret *et al.*, 1972). As far as we know, however, haemopoietic tumour cells in humans have never been investigated for receptors of vinca alkaloids. Our results demonstrate that the number of vindesine receptor sites differ from cell line to cell line, though the affinity does not markedly differ. It is interesting that MOLT-4 cells, established from acute lymphocytic leukaemia, and K562 cells, established from blastic crisis of chronic myelogenous leukaemia, had more receptor sites than HL-60 cells, established from acute promyelocytic leukaemia and normal blood lymphocytes. It remains to be determined, however, whether the difference in the number of receptor sites could explain the difference in clinical response to vinca alkaloids. We are now studying whether cells from leukaemic patients have vinca-alkaloid receptors and whether the presence of receptors is relevant to the effects of vinca-alkaloid therapy.

The authors wish to thank Dr T. Tsushima for helpful discussions and expert advice on the experimental procedures. This work was partially supported by a research grant from the Japanese Ministry of Education.

### REFERENCES

CHIRIFE, A. M. & STUDZINSKI, G. P. (1978) Definition of the cell cycle segment with special sensitivity to vinblastine. *Proc. Soc. Exp. Biol. Med.*, **157**, 206.

CUTTS, J. H. (1961) The effect of vincaleukoblastine on dividing cells *in vivo*. *Cancer Res.*, **21**, 168.

GOUT, P. W., VIJSCIK, L. L. & BEER, C. T. (1978) Difference between vinblastine and vincristine in distribution in the blood of rats and binding by platelets and malignant cells. *Eur. J. Cancer*, **14**, 1167.

KNIGHT, W. A., LIVINGSTONE, R. B., GREGORY, E. J. & MCGUIRE, W. L. (1977) Estrogen receptor as an independent prognostic factor for early recurrence in breast cancer. *Cancer Res.*, **37**, 4669.

MCGUIRE, W. L. (1975) Current status of estrogen receptors in human breast cancer. *Cancer*, **36**, 638.

MARANTZ, R., VENTILLA, M. & SHELANSKI, M. (1969) Vinblastine-induced precipitation of microtubule protein. *Science*, **165**, 498.

MILLER, T. P., JONES, S. E., CHESTER, A. B. & DORR, R. T. (1980) Phase II trial of vindesine in breast cancer, lymphoma and other tumors: Future directions. *Cancer Treat. Rev.*, **7** (Suppl.), 81.

OWELLEN, R. J., DONIGIAN, D. W., HARTKE, C. A., DICKERSON, R. M. & KUHAR, M. J. (1974) The binding of vinblastine to tubulin and to particulate fractions of mammalian brain. *Cancer Res.*, **34**, 3180.

OWELLEN, R. J., DONIGIAN, D. W., HARTKE, C. A. & HAINS, F. O. (1977) Correlation of biologic data with physicochemical properties among the vinca alkaloids and their congeners. *Biochem. Pharmacol.*, **26**, 1213.

OWELLEN, R. J., OWENS, A. H., JR, & DONIGIAN, D. W. (1972) The binding of vincristine, vinblasttine and colchicine to tubulin. *Biochem. Biophys. Res. Comm.*, **47**, 685.

PALMER, C. G., LIVENGOOD, D., WARREN, A. K., SIMPSON, P. J. & Johnson, I. S. (1960) The action of vincaleukoblastine on mitosis *in vitro*. *Exp. Cell Res.*, **20**, 198.

SECRET, C. J., HADFIELD, J. R. & BEER, C. T. (1972) Studies on the binding of $^3$H-vinblastine by rat blood platelets *in vitro*. *Biochem. Pharmacol.*, **21**, 1609.

STAMBAUGH, J. E., JR (1980) Phase II trial of vindesine in advanced neoplastic disease. *Cancer Treat Rev.*, **7** (Suppl.), 75.

WILSON, L., BAMBURG, J. R., MIZEL, S. B., GRISHAM, L. M. & CRESWELL, K. M. (1974) Interaction of drugs with microtubule proteins. *Fed. Proc.*, **33**, 158.

YOUNG, C. W. (1980) Vindesine trials at Memorial Sloan-Kettering Cancer Center. *Cancer Treat. Rev.*, **7** (Suppl.), 53.